# Microtron electron beam enables post-synthetic defect engineering in ultrasmall ceria nanocrystals

**DOI:** 10.1039/d6na00191b

**Published:** 2026-06-02

**Authors:** Zuzana Šiška, Tereza Sojková, Martin Sojka, Pavla Roupcová, Marián Mihálik, Kristýna Bukvišová, Dileep Krishnan, Lucie Šimoníková, Roman Gröger, Naděžda Pizúrová

**Affiliations:** a Institute of Physics of Materials and CEITEC IPM, Czech Academy of Sciences Žižkova 513/22 616 00 Brno Czech Republic sojkova@ipm.cz; b Central European Institute of Technology - CEITEC, Brno University of Technology Purkyňova 656/123 612 00 Brno Czech Republic; c Institute of Experimental Physics, Slovak Academy of Sciences Watsonova 47 Košice 040 01 Slovakia; d Thermo Fisher Scientific Brno Vlastimila Pecha 1282/12 627 00 Brno Czech Republic; e Thermo Fisher Scientific Achtseweg Noord 5 5651 GG Eindhoven The Netherlands; f Masaryk University, Department of Chemistry Brno Kotlářská 267/2 611 37 Czech Republic

## Abstract

This work explores high-dose MeV beam irradiation as a dopant-free, post-synthetic route to tune defect-related properties in 2–3 nm colloidal CeO_2_ nanoparticles. Oleate/oleylamine-stabilised nanoceria were reproducibly prepared *via* degassing-controlled thermal decomposition in dibenzyl ether. After that, the CeO_2_ nanoparticles were irradiated with a 16.5 MeV beam for 10, 40, and 80 min with nominal absorbed doses up to 171 ± 51 MGy while retaining crystalline fluorite cores. XPS and TEM-EELS analyses indicate the presence of irradiation-induced Ce^3+^/oxygen vacancy states, although spectral limitations prevent robust quantitative ranking of Ce^3+^. Surface-sensitive readouts show a non-monotonic response: the apparent optical bandgap narrows at the intermediate dose and partially recovers at the highest dose, accompanied by corresponding changes in the Urbach tail. In contrast, room-temperature magnetisation is substantially enhanced relative to pristine nanoceria and changes only weakly between the two highest-dose conditions. These observations suggest that defect centres persist within the nanoparticle volume even when the near-surface microstructure changes.

## Abstract 2

Electron-beam irradiation of ultrasmall CeO_2_ nanoparticles induces surface oxygen ejection and generates Ce^3+^ defects, enabling structure engineering and property adjustment.

## Graphical abstract

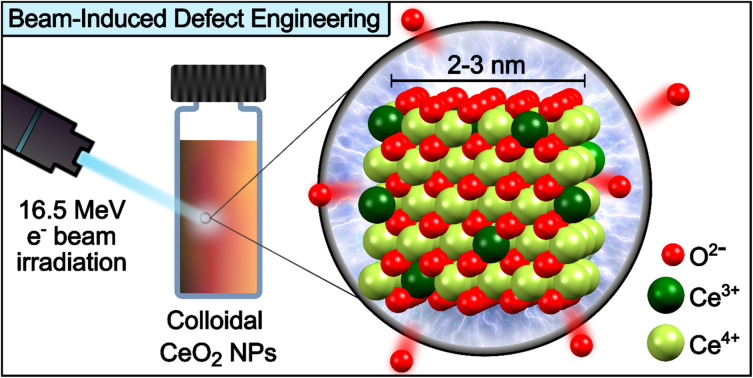

## 1. Introduction

Ultrasmall cerium oxide NPs have attracted significant attention in catalysis, nanomedicine, radiation protection, and spintronics. This profound utility is primarily derived from the redox flexibility of the cerium cation, which can reversibly transition between 4f^0^ (Ce^4+^) and paramagnetic 4f1 (Ce^3+^) electronic states, accommodating substantial oxygen non-stoichiometry without disrupting the host crystal lattice.^1–3^

Defect engineering allows tuning the defect-sensitive physicochemical properties of oxide nanomaterials while retaining their morphology and phase composition.^4^ In ultrasmall oxide NPs (≤5 nm), the high surface-to-volume ratio amplifies surface and near-surface defect chemistry, which makes vacancy populations and the local coordination environment dominant factors in property tuning.^4–6^ The size effect induces crystal expansion, attributed to a combination of increased concentrations of Ce^3+^ centers with larger ionic radii and lattice distortion. The expanded lattice facilitates the formation of oxygen vacancies due to the lower energy required for smaller sizes.^7–9^ CeO_2_ governed by oxygen vacancies and the Ce^3+^/Ce^4+^ couple^5,6,10^ enable tuning of catalytic and redox activity^5,6,11^ as well as optical properties, electronic structure (such as band edge shifts and sub-bandgap absorption),^12,13^ defect-induced magnetism (DIM),^14,15^ and bioactivity.^16,17^ In this size regime, these defects give rise to localized 4f states that can be described within a polaron model, providing the microscopic origin of these property changes. Both the electron localisation and local lattice distortion significantly affect optical properties, charge transport, and magnetic response.^18–20^

The optical response of cerium oxide nanoparticles (CONPs) is controlled by their narrow band gap (*E*_g_ ≈ 2.85–3.18 eV) compared to bulk CeO_2_, which is a wide band-gap semiconductor (*E*_g_ ≈ 3.33 eV).^21,22^ Such band-gap narrowing can shift the apparent absorption onset from the ultraviolet to the visible region.^23^ Defects introduce sub-gap energy levels that lead to the broadening of the absorption tail (Urbach tail) and an apparent narrowing of the optical band gap. Optical transitions in this regime primarily involve O 2p → Ce 4f defect states, as well as 4f–5d transitions modified by the presence of defects. These transitions involve charge transfer between oxygen and cerium sites, reflecting the hybridization of O 2p and Ce 4f orbitals which links the optical response to the redox-active Ce^3+^/Ce^4+^ couple. Charge transport is mediated by a thermally activated hopping mechanism between localized Ce^3+^ centres (small polaron hopping), with its efficiency depending on the concentration and distribution of defects.^18–20^ In photo-activated contexts, this lowers the photon energy required to drive reactive oxygen species (ROS) such as superoxide (˙O_2_^−^) and hydroxyl radicals (˙OH). These processes are facilitated by the Ce^3+^/Ce^4+^ redox couple which promotes adsorption by oxygen vacancies and enhances their catalytic performance.^24,25^

Nanoceria exhibits weak ferromagnetic-like signals at room temperature, most often associated with oxygen vacancies and paramagnetic Ce^3+^ centres, attributing the DIM to localized 4f^1^ electrons near oxygen vacancies. In contrast, Ce^4+^ (4f^0^) has all electrons paired and is diamagnetic. Localised 4f^1^ electrons carry magnetic moments, and in the presence of oxygen vacancies, they can lead to the emergence of DIM response, while an increasing concentration of Ce^3+^ centres typically manifests as an increase in saturation magnetization. This effect is often interpreted within models of defect-mediated exchange interactions, where localised electrons bound to vacancies (*e.g.*, F-centers) enable magnetic coupling between Ce^3+^ centres. Magnetic characterization can, therefore, serve as an indirect probe of irradiation-modified defect populations in CONPs.^12,15,26–28^

From a synthetic perspective, preparing ultrasmall ceria as a colloidally stable and reproducible model system remains nontrivial.^5–7^ Several liquid-phase routes yield CeO_2−*X*_ colloids, but achieving narrow 2–3 nm size distributions in ligand-stabilised systems requires carefully controlled high-temperature non-aqueous protocols.^29^ In β-diketonate-based thermal decomposition, reaction parameters such as the degassing step strongly influence nucleation and final particle properties, thereby determining nanoparticle size and resulting in a defect structure as well as the Ce^3+^/Ce^4+^ balance in ultrasmall ceria.^1,2,29–31^

Here, we implement reduced-pressure degassing during cerium precursor-based thermal decomposition in dibenzyl ether solvent. This procedure yields monodisperse 2–3 nm oleate/oleylamine-stabilised CeO_2_ nanocrystals, which provide a robust platform for post-synthetic modification.

Many post-synthetic modification methods use thermal treatments, chemical agents, and doping by other metals, which can unintentionally change particle size, crystallinity, or surface chemistry.^31^ Electron beam irradiation (EBI) offers a reagent-free route to generate defects at or near room temperature.^32,33^ When applied after synthesis, it can decouple defect formation from particle growth, thus preserving ultrasmall size and structural stability.^34^ Nevertheless, upon irradiation, slight growth or coalescence of nanoparticles may occur, although the extent of this process is limited in colloidal systems by the presence of surface-bound stabilizing molecules. Partial disruption of this layer can nevertheless lead to a slight increase in particle size and local reorganization of crystalline domains, giving rise to interfaces between regions with different structural arrangements. These interfaces are associated with lattice mismatch and induce local lattice distortions (lattice strain), which may affect the distribution of defects, while partially disordered interfacial layers result in an inhomogeneous, partially random distribution of defects both within the nanoparticle volume and at their surface.^35,36^ At high absorbed doses, competing processes such as defect creation *versus* partial healing, or re-oxidation, may occur. In some cases, irradiation-driven structural rearrangement or coarsening leads to complex, even non-monotonic dose responses.^32,37–42^ Nevertheless, systematic studies directly linking microtron parameters to structural and property changes in ultrasmall nanocrystals remain scarce. Most reports focus on nanopowders or kGy-level doses, while ligand-stabilised ultrasmall nanocrystals at very high doses are rarely explored.^43–45^

In this work, as-synthesised CeO_2_ nanocrystals in the range of 2–3 nm (denoted as CONPs_0) were subjected to controlled EBI using a microtron. The irradiated samples were analysed using several characterisation techniques and compared with the unirradiated sample CONPs_0. The influence of defect modifications caused by the MeV beam on the optical and magnetic properties was examined using structural (TEM and XRD), spectroscopic (XPS, EELS, and UV-vis), and magnetic (SQUID) characterization techniques to correlate processing conditions with material response. The results demonstrate the extent to which MeV irradiation affects the intrinsic oxygen vacancy states and the Ce^3+^/Ce^4+^ balance, while preserving the overall fluorite-dominant crystalline cores, despite a modest increase in mean particle size at higher irradiation exposure.

## 2. Experimental

### 2.1. Materials

Cerium(iii) acetylacetonate hydrate (Ce(acac)_3_ × H_2_O) was purchased from Alfa Aesar. Oleic acid (OA, 90%) was obtained from Lach-Ner, while oleylamine (OLAM, >70%) and dibenzyl ether (DBE) were purchased from Sigma-Aldrich. 1,2-hexadecanediol (98%) was obtained from TCI Chemicals. Acetone, absolute ethanol, and hexane were obtained from Penta Chemicals.

### 2.2. Synthesis of cerium oxide nanoparticles

**Fig. 1:**
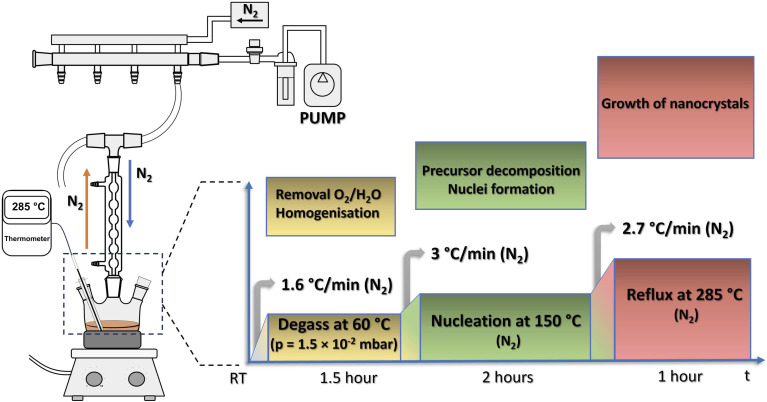
Schematic representation of the experimental setup (left) and the three-step synthesis procedure (right), including degassing under N_2_/vacuum, controlled heating, nucleation at 150 °C, and growth at 285 °C under reflux conditions, together with a simplified illustration of precursor transformation during the reaction.

In a typical synthesis, 2 mmol of Ce(acac)_3_ × H_2_O, 10 mmol of 1,2-hexadecanediol, 6 mmol of OA, and 6 mmol of OLAM were dissolved in 20 mL of DBE in a 50 mL three-neck round-bottom flask. The flask was equipped with an Allihn condenser and a magnetic stirrer and was connected to a Schlenk line for vacuum/nitrogen control. The solution was stirred continuously at 550 rpm throughout the synthesis. The synthesis is shown in Fig. 1, showing the experimental setup and the synthesis scheme in three main steps. In the degassing step, the solution was heated to 60 °C at 1.6 °C min^−1^ and maintained under reduced pressure (1.5 × 10^−2^ mbar) for 1.5 h to remove dissolved gases and residual moisture. In the nucleation step, the mixture was heated to 150 °C at 3 °C min^−1^ and held for 2 h to initiate nuclei formation. Finally, in the growth step, the solution was brought to reflux at 285 °C and maintained for 1 hour to promote controlled crystal growth. The reaction mixture was then cooled to room temperature under a nitrogen atmosphere.

The resulting yellow-brown product was subjected to washing and purification steps. The product was washed using 5 mL of hexane, 5 mL of acetone, and ethanol to a total volume of 45 mL, followed by centrifugation at 6000 rpm for 10 min. The washing step was repeated three times. To prevent aggregation during washing, all steps were carried out below 25 °C in an ultrasonic bath. The purified sample CONPs_0 was redispersed in 10 mL of hexane and filtered through a hydrophilic PTFE injection filter (25 mm diameter membrane disc with 0.45 µm pore size) prior to further use, where the resultant concentration of Ce reached 0.11 g L^−1^.

### 2.3. Surface modification using microtron irradiation

Three aliquots of the as-synthesised CONPs_0 sample were exposed to a pulsed 16.5 MeV electron beam in the MT25 microtron operated by the Nuclear Physics Institute, Czech Academy of Sciences, Řež. The accelerator was operated at a mean current of 10 µA (pulse length ≈ 3.5 µs; repetition rate 423 Hz). The irradiation times were 10 min (sample CONPs_10), 40 min (sample CONPs_40), and 80 min (sample CONPs_80). Immediately after treatment, all samples with Ce concentrations of 0.10–0.11 g L^−1^ were sealed and stored at low temperature to preserve radiation-induced surface and structural changes until further analysis.

#### 2.3.1 Beam diagnostics and dose estimation

Behind the 100 µm Al exit window, the beam was collimated to 6 ± 2 mm, fully covering the inner diameter (8 mm) of the glass vials. The second beam monitor registered integrated charges of 23.9 mC (40 min) and 45.1 mC (80 min). The integrated charge for the 10-minute run was not measured, but (based on proportional scaling) it is estimated to be 6.0 mC. With a spot area of 0.50 ± 0.16 cm^2^, these charges give fluences of 7.5 × 10^16^ e^−^ cm^−2^ (10 min), 3.0 × 10^17^ e^−^ cm^−2^ (40 min), and 5.6 × 10^17^ e^−^ cm^−2^ (80 min). Using the NIST-ESTAR mass-collision stopping power for 16 MeV electrons in *n*-hexane (*S*/*ρ* ≈ 1.9 MeV cm^2^ g^−1^), the corresponding estimated absorbed doses are 23 ± 7 MGy, 91 ± 27 MGy, and 171 ± 51 MGy, respectively. The quoted uncertainties reflect the ±2 mm collimation tolerance. Because the electron range in the liquid (∼8 cm) exceeds the sample thickness (∼0.8 cm) by an order of magnitude, geometry-dependent corrections are <5%. Direct alanine or radiochromic-film dosimetry was not available during this pilot study. Owing to ±30% uncertainty in the estimated absorbed doses, the irradiation time series (10, 40, and 80 min) should be treated as an ordered sequence of increasing nominal dose rather than as a quantitatively calibrated dose scale. Future work will implement direct dosimetry to refine absolute dose values and enable more rigorous dose–response analysis.

### 2.4. Characterisation

#### 2.4.1 Transmission electron microscopy (TEM)

Detailed structural characterisation of the CONP samples was performed using a JEOL JEM-2100F transmission electron microscope operated at an accelerating voltage of 200 kV and a Titan Themis 60–300 TEM operated at 300 kV. Samples were prepared by drop-casting a dilute dispersion of CONPs onto holey carbon films supported on 300-mesh copper grids, followed by solvent evaporation at room temperature. Micrographs were processed using TIA (Thermo Fisher Scientific) and DigitalMicrograph (Gatan).

#### 2.4.2 Powder X-ray diffraction (PXRD)

PXRD was performed to determine the phase composition of CONPs_0. The XRD pattern was recorded using an Empyrean diffractometer (Malvern Panalytical) equipped with Co Kα radiation (*λ* ≈ 1.79 Å), operating in Bragg–Brentano geometry over the 2*θ* range of 15–115°, with a step size of 0.04°. A dispersion of CONPs_0 was drop-cast onto a zero-background silicon substrate and allowed to dry at room temperature. Data were analysed using the software HighScore Plus 4.0.

#### 2.4.3 Dynamic light scattering (DLS)

Dynamic light scattering was used to determine the hydrodynamic size and polydispersity index (PDI) and to assess the potential formation of aggregates in the CONP dispersions. Measurements were performed using a Zetasizer Ultra (Malvern Panalytical). The scattered intensity of CONPs dispersed in organic solvent was collected using Multi-Angle DLS (MADLS) at three detection angles: 13°, 90°, and 173°, with a 632.8 nm laser source. All measurements were conducted at room temperature after instrument calibration. For each sample, three measurements were conducted with the mean ± standard deviation reported in Table 1.

**Table 1:** Mean TEM core diameters (*d*_TEM_) and DLS hydrodynamic diameters (*D*_H_; intensity-weighted) for CONPs_0/10/40/80. The values are given as mean ± SD (standard deviation). *n*_TEM_ is the number of NPs analysed by TEM

Sample	*n*_TEM_	*d*_TEM_ ± SD(nm)	*D*_H_ (nm)
CONPs_0	300	2 ± 0.6	4 ± 1
CONPs_10	300	2 ± 0.6	5 ± 2
CONPs_40	200	3 ± 0.9	8 ± 4
CONPs_80	200	3 ± 0.9	10 ± 2

#### 2.4.4 Elemental analysis

For elemental analysis, 100 µL of each sample was dried under vacuum for 24 h, dissolved in aqua regia, and then diluted to 25 mL. The concentration of CONPs was determined using an inductively coupled plasma optical emission spectrometer (ICP-OES) iCAP PRO XPS (ThermoFisher Scientific).

#### 2.4.5 X-ray photoelectron spectroscopy (XPS)

The chemical compositions of the CONP samples were analysed using a Kratos AXIS Supra X-ray photoelectron spectrometer under ultra-high vacuum conditions, with a monochromatic Al Kα (1486.7 eV) excitation source. Survey spectra were collected to determine the overall elemental composition. High-resolution spectra were acquired for the Ce 3d and O 1s core levels to evaluate the oxidation state of cerium and the chemical environment of oxygen, respectively.

#### 2.4.6 Electron energy loss spectroscopy (EELS)

The oxidation state was determined using an Iliad 300 TEM instrument (ThermoFisher Scientific), operating at an acceleration voltage of 300 kV without a monochromator. The EELS spectra were acquired from a cluster of NPs with a dispersion of 0.42 eV per channel and were analysed using Hyperspy.^46^

#### 2.4.7 UV-vis spectrophotometry

Optical absorption spectra were recorded using a Jenway 7415 Nano micro-volume spectrophotometer. Samples diluted in ethanol were measured in a quartz cuvette with 1 cm path length. Spectra were collected in air at room temperature over the wavelength range of 198–1000 nm. The measured absorbance *A*(*λ*) was converted to an effective attenuation coefficient using the Beer–Lambert law *α*(*λ*) = 2.303 *A*(*λ*)/*l* with *l* = 1 cm and analysed in photon-energy units (*hν*). The apparent optical gap (*E*_g_) was estimated from Tauc-type plots by extrapolating the linear region of (*αhν*)^1/2^*vs. hν* to the energy axis. For completeness, the detailed analyses using the Tauc model together with the Urbach-tail fitting procedure are provided in the SI (Sec. S3.6 and Fig. S5–S7).

#### 2.4.8 Magnetic measurements (SQUID)

Magnetic measurements were performed using a superconducting quantum interference device (SQUID) magnetometer (Quantum Design). Magnetic hysteresis loops were recorded at 300 K after demagnetisation from both the negative and positive field directions. The magnetic field was swept between ±7 T (equivalent to ±70 kOe, *i.e.*, ±5.57 MA m^−1^). Samples were prepared by directly weighing the dried powders into the sample capsule.

## 3 Results and discussion

### 3.1 Synthesis modification

The thermal decomposition method was selected because it reliably yields ultrasmall crystalline NPs with narrow size distributions.^30^ The cerium precursor was decomposed in DBE at high temperature in the presence of OA and OLAM, which act as coordinating ligands. Phenyl ether (b.p. 260 °C) was initially tested as the solvent, but no crystalline CONPs formed under these conditions. In contrast, switching to DBE (b.p. 298 °C) led to reproducible formation of CeO_2_ nanocrystals. However, at temperatures close to 300 °C, benzyl alcohol, benzaldehyde, and toluene were formed as by-products.^30,47,48^ Therefore, the reflux temperature (controlled growth) was limited to 285 °C to ensure stable reaction conditions while avoiding premature decomposition of the solvent.

The size control was governed by a precursor:ligand ratio of 1 : 6, consistent with previous reports on sub-5 nm CONPs.^49^ The three-step synthesis used here (degassing, nucleation, and growth in Fig. 1) was adapted from established thermal decomposition protocols.^48^ An extra emphasis was put on the degassing stage. Degassing at 60 °C under reduced pressure (1.5 × 10^−2^ mbar) for 1.5 h efficiently removed dissolved gases and residual moisture. This step minimised bubbling of the reaction mixture into the condenser and prevented changes in its composition. This adjustment reproducibly yielded monodisperse 2–3 nm CONPs and was maintained as a key modification relative to standard procedures.^48,50,51^

Following degassing, the mixture was heated to 150 °C at 3 °C min^−1^ (above the 131–132 °C melting point of the precursor). The melting point of the precursor was used as an approximate thermal reference for the subsequent nucleation step. As the compound is a hydrate, this transition does not correspond to melting but rather to dehydration and partial decomposition of the complex. Taking these factors into consideration, heating the degassed reaction mixture to 150 °C ensures sufficient precursor decomposition, leading to the formation of reactive species required for nucleation, and it was held for 2 h to generate a sufficient population of stable crystal nuclei.^52–54^ The temperature was then increased to 285 °C, within the thermal stability range of DBE, which enabled controlled growth of the pre-formed nuclei while minimising secondary nucleation. The selected reaction time of 1 h ensured sufficient particle growth without excessive growth and broadening of the size distribution of CONPs_0.^15,55^

### 3.2 Post-synthesis microtron irradiation

High-energy EBI was chosen as a reagent-free approach to tailor defect populations in ceria, whose physical and redox behaviour allows controlling optical and DIM response.^4,13,15,25,56^ To introduce defects in a controlled manner, a pulsed 16.5 MeV microtron beam was applied at nominal absorbed doses up to 171 ± 51 MGy. To the best of our knowledge, this regime has not been systematically investigated for post-synthetic treatment of CONPs. At these energies, elastic scattering generates knock-on defects, such as Frenkel pairs, defect clusters in the oxygen sublattice, and Ce vacancies, which are difficult to achieve by irradiation at lower energies. In parallel, inelastic processes induce surface radiolysis, including Ce–O bond breaking, ligand scission, and even partial Ce^4+^ → Ce^3+^ reduction.^33,57^

Under the applied geometry, electron beam-induced heating is expected to be minimal. However, at high absorbed doses, localised energy deposition may contribute to subtle changes in nanoparticle size and the surface structure. In parallel, irradiation-driven processes such as vacancy recombination or reoxidation cannot be excluded, as they may reduce the population of oxygen vacancies and promote partial back-conversion of Ce^3+^ to Ce^4+^ at the surface, leading to partial recovery of defect states.^38,39,42,58^

### 3.3. Structural and phase analysis

**Fig. 2:**
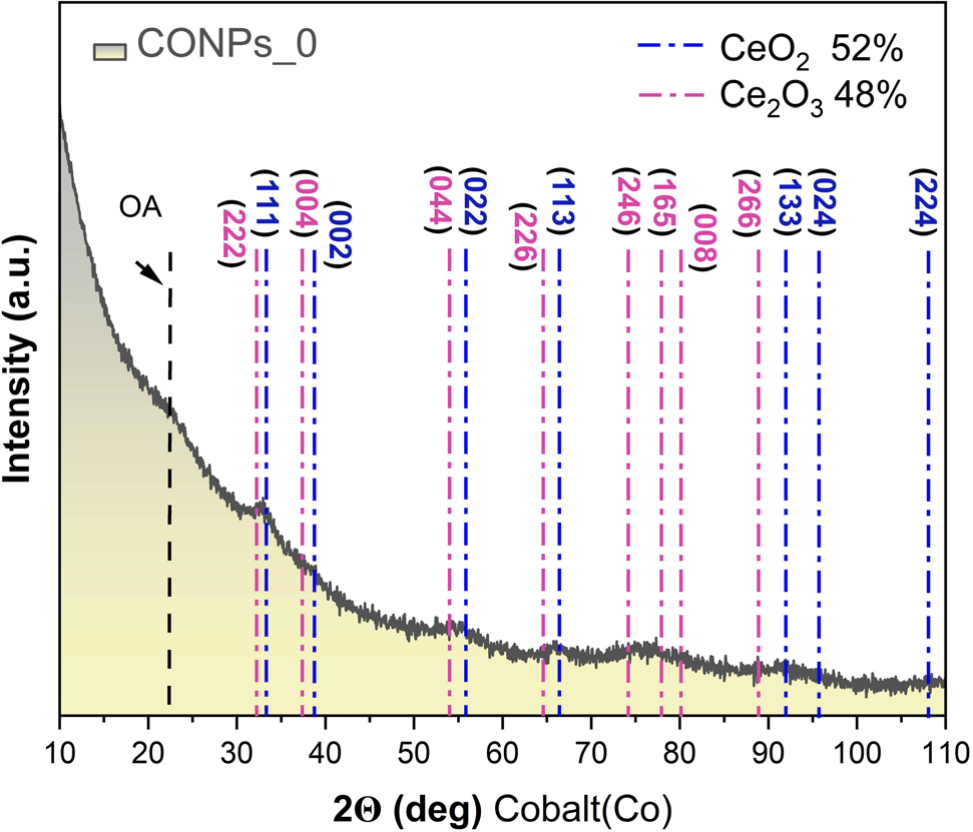
The XRD pattern of CONPs_0 obtained immediately after synthesis. The characteristic reflections show a presence of fluorite-type CeO_2_ (depicted by dashed-dot blue lines) and bixbyite-type Ce_2_O_3_ (depicted by dashed-dot pink lines), yielding phase fractions of 52% and 48%, respectively. A weak reflection is observed at 22.5° and suggests the presence of the OA surfactant at the NP surface.

**Fig. 3:**
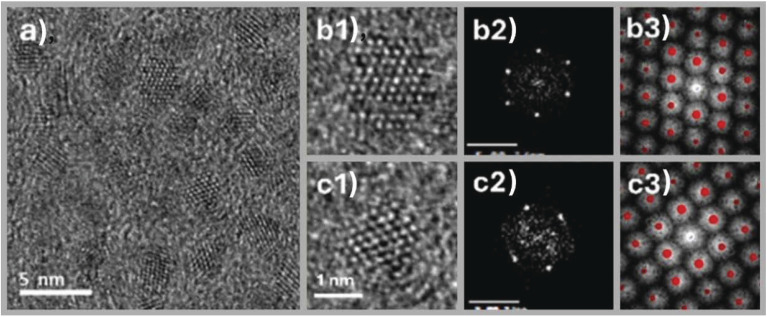
TEM characterisation of the CONPs_0 sample. (a) TEM micrograph showing a dense population of ultrasmall NPs. HR-TEM images of two representative particles (b1 and c1) reveal clear lattice fringes and irregular faceting. The corresponding FFT patterns (b2 and c2), together with their evaluation using JEMS software (b3 and c3), confirm the fluorite CeO_2_ structure with a [101] zone axis for both particles.

Immediately following synthesis, the CONPs_0 sample was characterised by high-resolution TEM (HR-TEM) and XRD to assess crystallinity and obtain structural information. XRD analysis in Fig. 2 performed on a purified sample immediately after cleaning to minimise contributions from organic residues reveals cerium oxide phase CeO_2_ with a fluorite structure (ICSD no. 98-002-8709) and bixbyite structure Ce_2_O_3_ (ICSD no. 98-062-1711). The Rietveld refinement indicates the coexistence of both phases with comparable contributions of 52% CeO_2_ and 48% Ce_2_O_3_ with estimated average crystallite sizes of 5 nm for CeO_2_ and 3 nm for Ce_2_O_3_. The reflections of the CONPs_0 pattern appear broader and less intense, which is attributed to the small nanoparticle size.^7^ A diffraction shoulder at 22.5° could indicate the possible presence of adsorbed OA as a capping ligand, consistent with previous studies on chemisorbed organic layers.^59–61^ TEM shows that the as-synthesized CONPs_0 sample consists of ultrasmall particles 2–3 nm in diameter with a narrow size distribution (Fig. 3), consistent with the optimised synthesis conditions. The smaller size determined by HR-TEM (2 ± 0.6 nm) reflects its local and particle-specific nature, whereas XRD provides volume-weighted average crystallite sizes for individual phases, with size values in the order of nanometers. HR-TEM images of two representative particles (Fig. 3b1 and 3c1) display well-resolved lattice fringes indicative of high crystallinity. The corresponding Fast Fourier Transform (FFT) patterns (Fig. 3b2 and 3c2), together with the filtered FFT images (Fig. 3b3 and 3c3), can be indexed to the fluorite CeO_2_ structure with a [101] zone axis for both particles.

**Fig. 4:**
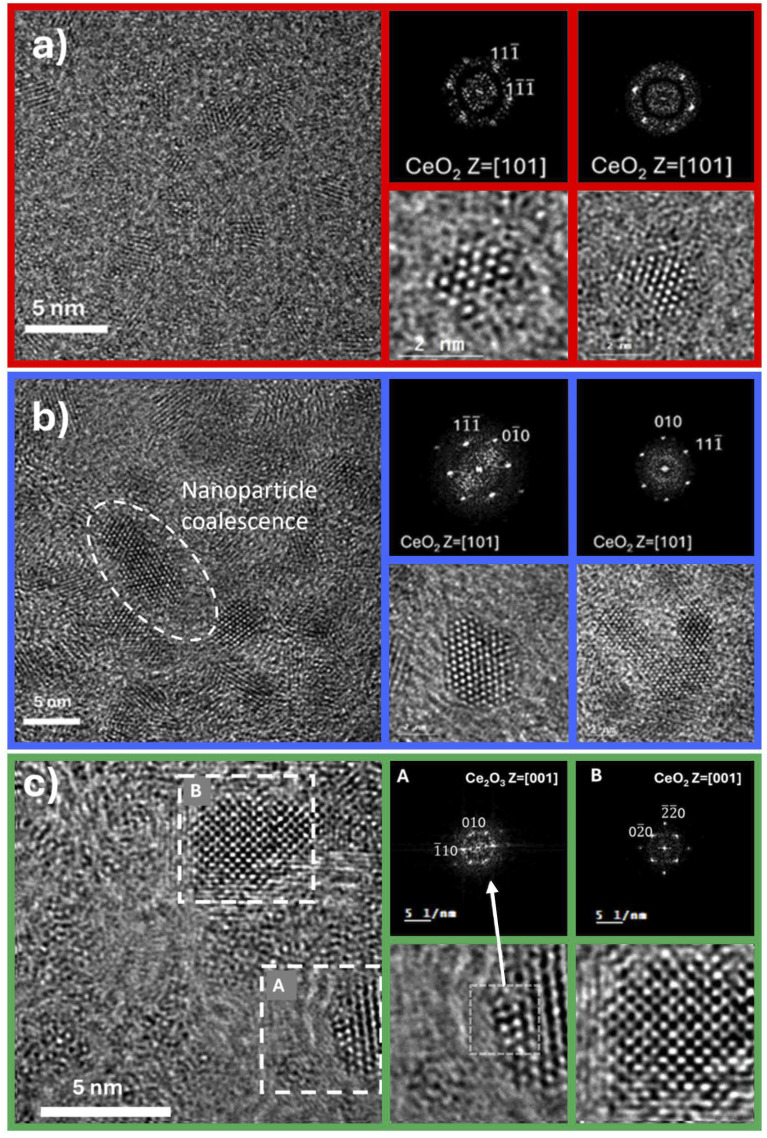
HR-TEM images of irradiated CONPs: (a) CONPs_10, (b) CONPs_40 (two coalesced nanoparticles highlighted), and (c) CONPs_80. Insets: FFT patterns confirming the fluorite-type CeO_2_ phase in all samples and showing that CONPs_80 remains highly crystalline. However, additional reflections from selected surface regions, particularly particle A in (c), are consistent with bixbyite-like (Ce_2_O_3_) ordering.

After 10 minutes of irradiation (CONPs_10, Fig. 4a), the NPs appear more irregular. Despite these morphological changes, the NP volumes remain crystalline, and no amorphous surface layers are observed. A slight increase in particle size is observed in the CONPs_40 sample (Fig. 4b), which we tentatively attribute to the coalescence of smaller primary NPs. At the same time, the particle shape becomes more regular, with more clearly defined crystalline facets. Even at the highest EBI dose (CONPs_80, Fig. 4c), the NPs remain crystalline. FFT analysis of the HR-TEM images confirms the fluorite-type CeO_2_ structure throughout the particle volumes. The additional reflections from selected surface regions of the CONPs_80 sample, particularly particle A (Fig. 4c), are consistent with bixbyite-like (Ce_2_O_3_) ordering. Since CeO_2_ and cubic Ce_2_O_3_ share an fcc cation sublattice and the structure of bixbyite can be considered a 2 × 2 × 2 fluorite supercell with ordered vacancies, the coexistence of these two phases is structurally plausible.^62,63^

### 3.4 Nanoparticle size analysis by TEM and DLS

**Fig. 5:**
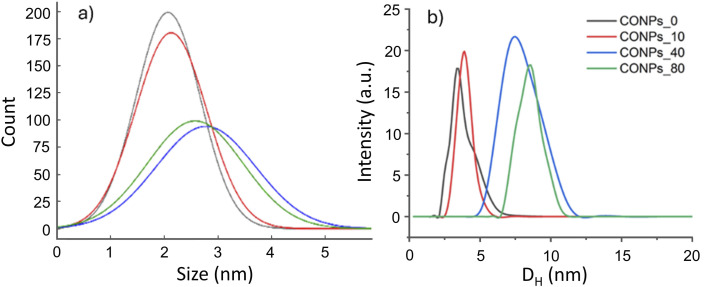
Size statistics of (a) HR-TEM images and (b) intensity-weighted hydrodynamic size (*D*_H_) histograms for the sample CONPs_0/10/40/80. The distribution size in (a) is fitted with Gaussians whose parameters (mean, SD) are summarised in Table 1.

Because the ultrasmall crystalline NPs are embedded in an organic matrix, automated image segmentation was unreliable. Their core diameters were therefore measured manually in TIA. In contrast to TEM/HR-TEM, which measures the crystalline core, DLS provides intensity-weighted hydrodynamic diameters in dispersion (Fig. 5), including the organic shell and any small aggregates. Accordingly, DLS values are interpreted here primarily as indicators of the colloidal state rather than as direct measures of core size. This is supported by the observation that DLS shows an increase in the hydrodynamic diameter, likely due to modifications of the ligand shell or the formation of small aggregates, whereas TEM reveals only a slight increase in the size of the crystalline cores. Number-weighted distributions (Table S1 and Fig. S1, SI) were also evaluated to enable a direct comparison with TEM. These values are lower than the corresponding intensity-weighted diameters, reflecting differences in the weighting of particle populations. Importantly, the dose-dependent increase in size remains consistent across all weighting methods. Nevertheless, DLS measures hydrodynamic diameters that include the organic ligand shell and solvation layer, and therefore, even number-weighted values do not directly correspond to the core sizes obtained by TEM.

The observed increase in particle size with irradiation time follows the increase in the nominal absorbed dose. A strictly linear relationship between particle size and dose is not expected. The increase in particle size is attributed to partial degradation of the organic ligand shell (oleate/oleylamine), which facilitates nanoparticle coalescence.^64^ At higher doses, however, further growth does not necessarily proceed proportionally, as the system may become limited by the availability and mobility of remaining primary particles after initial coalescence. This can result in a deviation from a simple linear trend.

PDI values close to 0.1 typically indicate a narrow size distribution.^65^ The pristine sample (CONPs_0) shows a PDI of 0.13. After irradiation, the PDI for CONPs_40 and CONPs_80 increases slightly to 0.18 and 0.16, respectively (Sec. S3.4, Table S1, SI), consistent with a modest broadening and/or changes in shell morphology.

### 3.5 XPS analysis of the oxidation state and defects

**Fig. 6:**
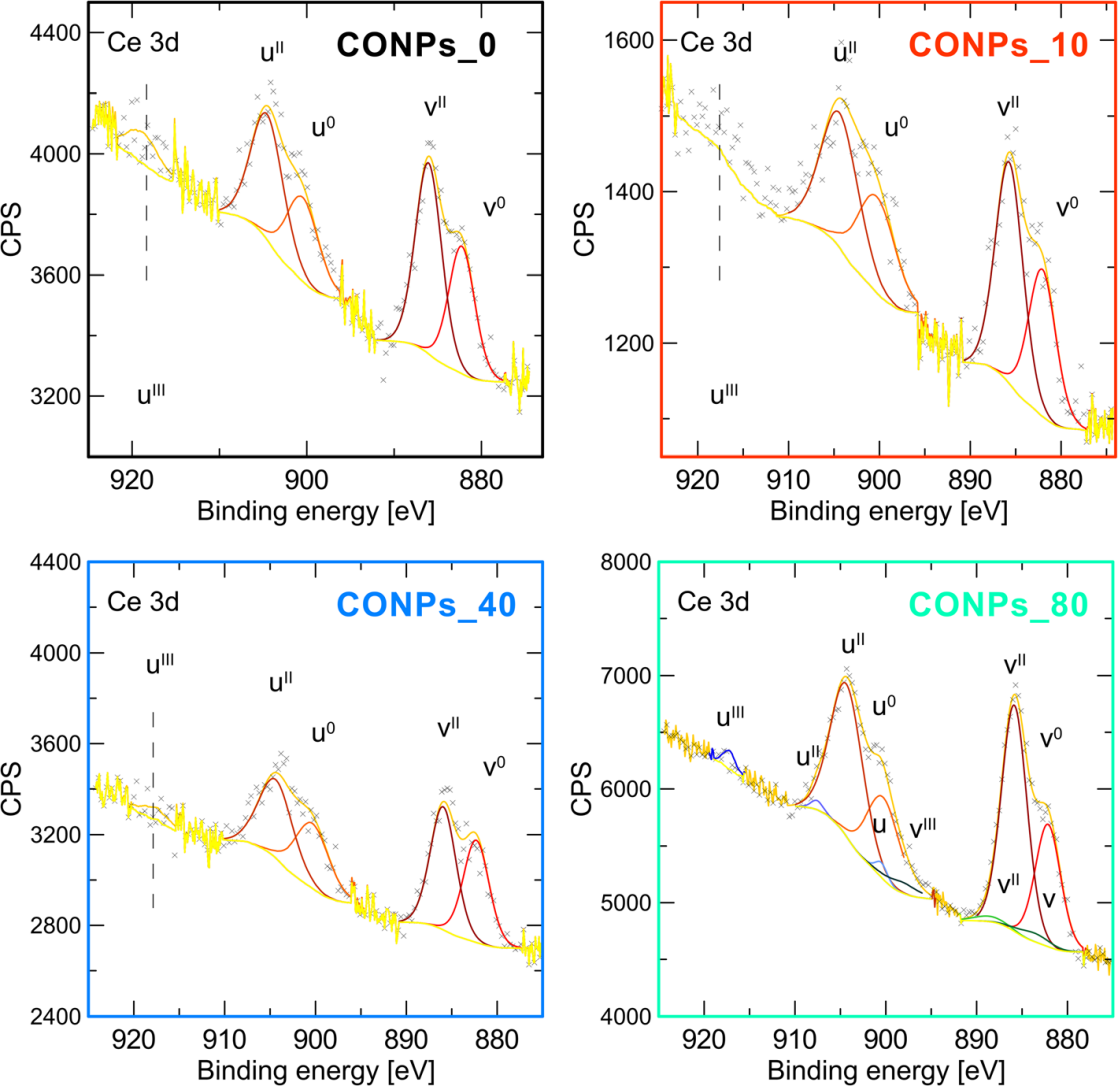
Ce 3d XPS spectra of CONPs_0/10/40/80 (yellow traces), with multiplet fits (coloured component curves). The spin–orbit-split Ce 3d_5/2_ (v) and Ce 3d_3/2_ (u) envelopes comprise contributions from Ce^3+^. CONPs_80 exhibits a Ce^4+^ contribution, which is interpreted as near-surface re-oxidation at the highest dose. This surface-specific observation should not be conflated with the bulk redox state probed by EELS and SQUID. Given the organic overlayer, trends are interpreted qualitatively.

**Fig. 7:**
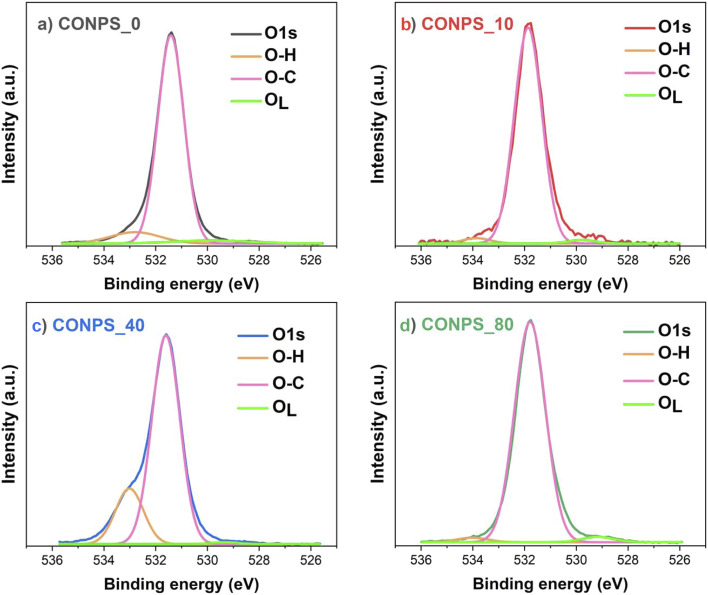
O 1s XPS spectra of (a) CONPs_0, (b) CONPs_10, (c) CONPs_40 and (d) CONPs_80 with three component fits: O_L_ (green) – a bulk-like lattice O^2−^ in CeO_2_ and SiO_2_ (528.8–529.8 eV); O–C (pink) – surface oxygen bound to C–O from OA and Ce^3+^-O (531.2–532.0 eV); O–H (yellow) – higher binding energy adsorbate species of OA or defects (532.0–533.5 eV).

XPS reveals a contribution of Ce^3+^ components within the Ce 3d envelope (Fig. 6a–d), superimposed on the spin–orbit split Ce 3d_5/2_ and Ce 3d_3/2_ series (Table S2, SI). In the Ce 3d XPS spectrum, a characteristic signal associated with Ce^4+^ is observed only in the CONPS_80 sample. In this CONPS_80 sample, the relative ratio of Ce^3+^/Ce^4+^ contributions was determined based on peak areas, with 93% Ce^3+^ and 7% Ce^4+^ detected. Because XPS probes only the outermost ∼1 nm of the nanoparticle, this is interpreted as partial re-oxidation or restructuring of the near-surface layer at the highest dose and does not necessarily reflect the redox state of the nanoparticle interior. In the O 1s spectra (Fig. 7a–d), the dominant central peak at ∼531.2–532.0 eV, here labeled O–C, is attributed to surface oxygen bonded to the C–O group of the OA surfactant, including Ce^3+^-O oxygen , while lattice oxygen (O_L_) of Ce^4+^ appears as a small peak at 528.8–529.8 eV. In addition to the Ce^4+^ contribution, the O_L_ intensity partially reflects contributions from Si^4+^, which are likely related to residues of silicone paste used to seal the joints of the Schlenk line during synthesis. Nevertheless, this assignment is consistent with previous XPS studies of nanoceria, where the intermediate binding energy component is associated with cerium-related and hydroxylated surface oxygen rather than with stoichiometric lattice O^2−^. An additional oxygen peak (O–H) is detected at 532.0–533.5 eV (Table S3, see the SI), with its highest intensity in CONPs_40 (Fig. 7c). This feature is most commonly attributed to surface hydroxyl groups, adsorbed water, or chemisorbed organic species,^66–68^ although Majumder *et al.*^69^ reported a correlation between the intensity of this component and the density of surface oxygen vacancy-related active centres. The work of Sehar *et al.*^70^ attributes the additional oxygen peak to hydroxyl or other defective sites, suggesting an increased number of active centres. These CONPs demonstrated enhanced dye degradation and antibacterial activity.^70^ The two interpretations are not mutually exclusive. Irradiation-induced vacancy formation may simultaneously increase hydroxyl adsorption at defect sites. In either case, the elevated O–H contribution in CONPs_40 is consistent with the highest surface defect density at the intermediate dose, as also reflected in the Urbach energy and apparent optical gap data.

These observations suggest that the surface defect structure is actively modified under irradiation and that this modification reflects the underlying chemical processes responsible for the observed redox changes. Electron irradiation of *n*-hexane is expected to generate reactive species, such as alkyl and hydrogen radicals,^71^ which can promote the reduction of Ce^4+^ to Ce^3+^, particularly at intermediate irradiation times. At higher irradiation doses, however, competing processes, such as partial reoxidation, may become significant, potentially due to residual oxygen in the system, either dissolved in the solvent or in the vial headspace.

The valence-band XPS spectra (Fig. S3 in SI) show that EBI exposure of samples leads to modest broadening of the valence region and additional intensity within approximately 2 eV of the Fermi level (*E*_F_), consistent with defect-related states within the band gap. However, due to attenuation by the organic ligand overlayer and the possibility of differential surface charging, the apparent valence band maximum (*E*_VB_) cannot be reliably referenced to the Fermi level and is therefore not used to derive the band gap estimate. The valence band spectra are presented qualitatively as supplementary evidence for irradiation-induced sub-gap state formation only.

**Fig. 8:**
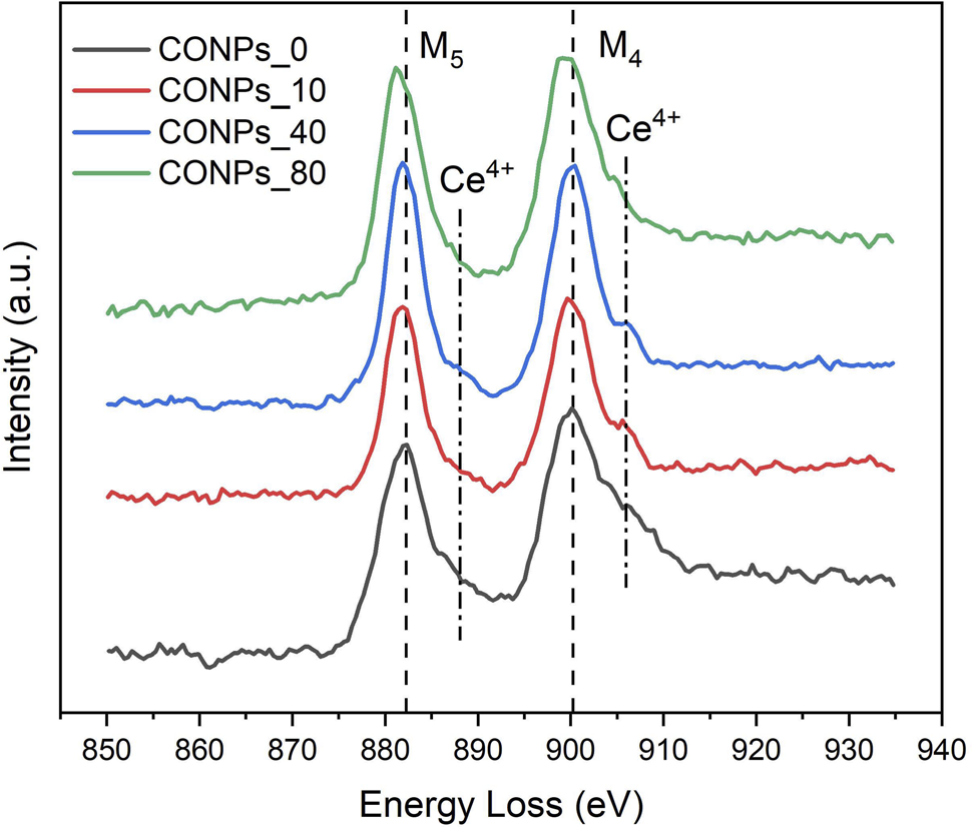
Qualitative changes in white-line shape and the M_5_/M_4_ branching ratio across the dose series. Ce^3+^-associated spectral features (low-energy shoulders at ∼883–885 eV and ∼901–902 eV and an increased M_5_/M_4_ ratio) are most apparent in CONPs_40, with partial attenuation in CONPs_80 consistent with near-surface reorganisation at the highest dose. The vertical dashed line indicates the reference Ce^3+^ M_4_,_5_ white-line positions.

Complementary TEM-EELS measurements at the Ce M_4/5_ edges (Fig. 8) provide qualitative evidence for irradiation-induced changes in the electronic structure of Ce, as reflected in the shape and relative intensity of the white lines (intense 3d → 4f transition peaks at the M_5_ and M_4_ edges). Quantitative M_5_/M_4_ branching ratio analysis is limited by the signal-to-noise ratio. The qualitative spectral evolution is nevertheless consistent with a partial increase in Ce^3+^ contribution. Therefore, we refrain from ranking the Ce^3+^ fraction within individual doses based solely on EELS. Nevertheless, with increasing EBI dose, the spectra qualitatively indicate an increased Ce^3+^ contribution within the probed nanoparticle volume, as observed in the XPS data. This suggests that the redox modification is not solely confined to the particle surface. The weak ELNES (Energy Loss Near Edge Structure) features consistent with Ce^3+^ appear on the low-energy side of the M_5_ and M_4_ edges (883–885 eV and 901–902 eV) relative to the Ce^4+^ white-line positions at 885 eV (M_5_) and 903 eV (M_4_).

Compared with CONPs_0, CONPs_10 shows a higher apparent M_5_/M_4_ edge ratio and an improved signal-to-noise ratio, indicating partial reduction. At the intermediate dose (CONPs_40), the spectral features associated with Ce^4+^ are most pronounced within the series: the M_5_/M_4_ branching ratio shift and the low-energy shoulder intensity are clearest in this sample. This is qualitatively consistent with the optical data, where CONPs_40 likewise exhibits the most pronounced gap narrowing and the highest Urbach energy, both indicative of the maximum near-surface defect density at an intermediate dose. For CONPs_80, a slight low-energy shift of the white lines for Ce^3+^ and the hint of shoulders corresponding to Ce^4+^ are visible, though these features appear less pronounced than in CONPs_40. This partial spectral recovery at the highest dose parallels the partial recovery of the optical gap and reduction in Urbach energy observed by UV-vis spectrophotometry and is consistent with near-surface reorganisation or partial re-oxidation suggested by XPS. Overall, the EELS spectral evolution is qualitatively consistent with the coexistence of Ce^4+^/Ce^3+^ ions across all samples.^72,73^ The nominal-dose-correlated increase in Ce^4+^ character appears most pronounced at the intermediate dose (CONPs_40) and is partially attenuated at the highest dose (CONPs_80). This non-monotonic pattern mirrors the optical response and supports the interpretation that surface defect density peaks at intermediate irradiation time. These conclusions are necessarily qualitative given the signal-to-noise constraints and the absence of monochromated EELS data.

The qualitative EELS trend – with Ce^3+^ signatures most pronounced at the intermediate dose and partially attenuated at the highest dose – establishes a spectroscopic basis for the non-monotonic optical response described in the following section and supports the interpretation that both phenomena reflect the same underlying evolution of the near-surface defect landscape rather than independent effects.

### 3.6. Optical properties

The evolution of the optical absorption with irradiation time is shown in the UV-vis spectra (Fig. S4, SI). All samples exhibit a pronounced absorption band at 232-239 nm, followed by a broad sub-gap tail extending into the near UV and visible region. The intense band in the UV region is commonly attributed to charge-transfer transitions, while the absorption onset is defined by the long-wavelength edge of this tail.

The Tauc plot was originally derived for amorphous semiconductors but has been widely applied to nanocrystalline oxide systems. It was previously applied to ultrasmall CeO_2_ nanoparticles to track band gap narrowing associated with size reduction and surface defect accumulation.^6,7,74^ In ultrasmall nanocrystals such as those studied here, the optical absorption is governed not only by the bulk band structure but also by quantum confinement, surface states, and a pronounced sub-gap tail arising from structural and chemical disorder at the particle surface. Under these conditions, the Tauc intercept does not strictly correspond to the fundamental band gap of bulk CeO_2_ but serves as a reproducible, comparative descriptor of the absorption onset. It is used here exclusively in this phenomenological sense to track relative shifts across the irradiation series, not to make absolute claims about the electronic band structure. The complete set of plots and details of fitting are provided in the SI (Fig. S5–S6).

From *E*_g_, the corresponding absorption-edge wavelength *λ*_e_ = *h*_c_/*E*_g_ (where *h*_c_ = 1240 eV nm) was calculated as a convenient descriptor of the absorption onset. The Urbach energy *E*_U_ was obtained from the exponential absorption tail in the sub-gap, where the details and goodness-of-fit metrics are given in the SI, Fig. S7.^75^ Together, optical parameters *E*_g_, *λ*_e_, and *E*_U_ are summarised in Table 2. The reference sample CONPs_0 appears to be influenced by disorder and surface defects, as well as by the presence of localised states within the bandgap. This manifests as a slightly reduced gap (*E*_g_ = 3.05 eV) and higher Urbach energy compared to bulk CeO_2_.^76^

**Table 2:** Summary of optical properties of the CONP series extracted from UV-vis spectra. The apparent optical gap *E*_g_ and Urbach energy *E*_U_ were obtained from the Tauc and Urbach analyses. The increase in *E*_U_ indicates enhanced structural disorder after irradiation. The *E*_U_ for CONPs_10 is slightly reduced compared to CONPs_0, which may reflect partial annealing of pre-existing surface disorder at low fluence, prior to net defect accumulation at higher doses

Sample	CONPs_0	CONPs_10	CONPs_40	CONPs_80
*E*_g_ (eV)	3.05	2.99	2.91	2.98
*λ*_e_ (nm)	407	415	426	416
*E*_U_ (eV)	0.73	0.69	0.78	0.67

A narrowing of the optical band gap under irradiation (30–90 kGy) was reported previously by Khan *et al.*^13^ and attributed to the increased concentration of vacancies and population of Ce^3+^ centres. In the present study, a similar trend is observed, where the sample irradiated for 40 min (CONPs_40) exhibits the lowest apparent optical gap in the series. However, given the ±30% uncertainty in the estimated absorbed doses, this observation is best described as a nominal-dose-dependent correlation rather than a precisely calibrated dose–response relationship. At the highest irradiation dose (CONPs_80), this trend partially reverses: a slight blue shift is observed, along with an increase in *E*_g_ and a decrease in *E*_U_. This partial recovery is consistent with the spectroscopic evidence for near-surface reorganisation at the highest dose, as independently supported by XPS and EELS, and is not attributable to dose uncertainty alone.

The observed changes in the band gap (including the red shift at the intermediate dose and its partial recovery at the highest dose) cannot be unambiguously attributed to quantum confinement, which would lead to a blue shift with decreasing particle size.^12^ In this system, a slight increase in nanoparticle size is observed, while the evolution of the band gap does not directly correlate with particle size. B. Choudhury and A. Choudhury^77^ proposed that the red shift in CeO_2_ nanoparticles may be related to an interfacial polaron effect associated with electron–phonon coupling, which can be enhanced with decreasing particle size. However, other studies show that polaronic effects are primarily governed by charge localization associated with defects (Ce^3+^ and oxygen vacancies), independent of particle size. While polaron transport can be thermally, luminescently, and chemically activated,^19,20^ the dominant factor determining the electronic structure in this case is the concentration of defects induced by irradiation. An increased fraction of defect states at the intermediate dose leads to band-gap narrowing, accompanied by an increase in the Urbach energy. In contrast, the partial recovery of optical parameters at the highest dose, accompanied by a reduced fraction of optically active defects, suggests a decrease in defect heterogeneity at higher reduction levels, which may indicate partial recombination or restructuring of defect states.^19,78^

### 3.7. Magnetic properties

**Fig. 9:**
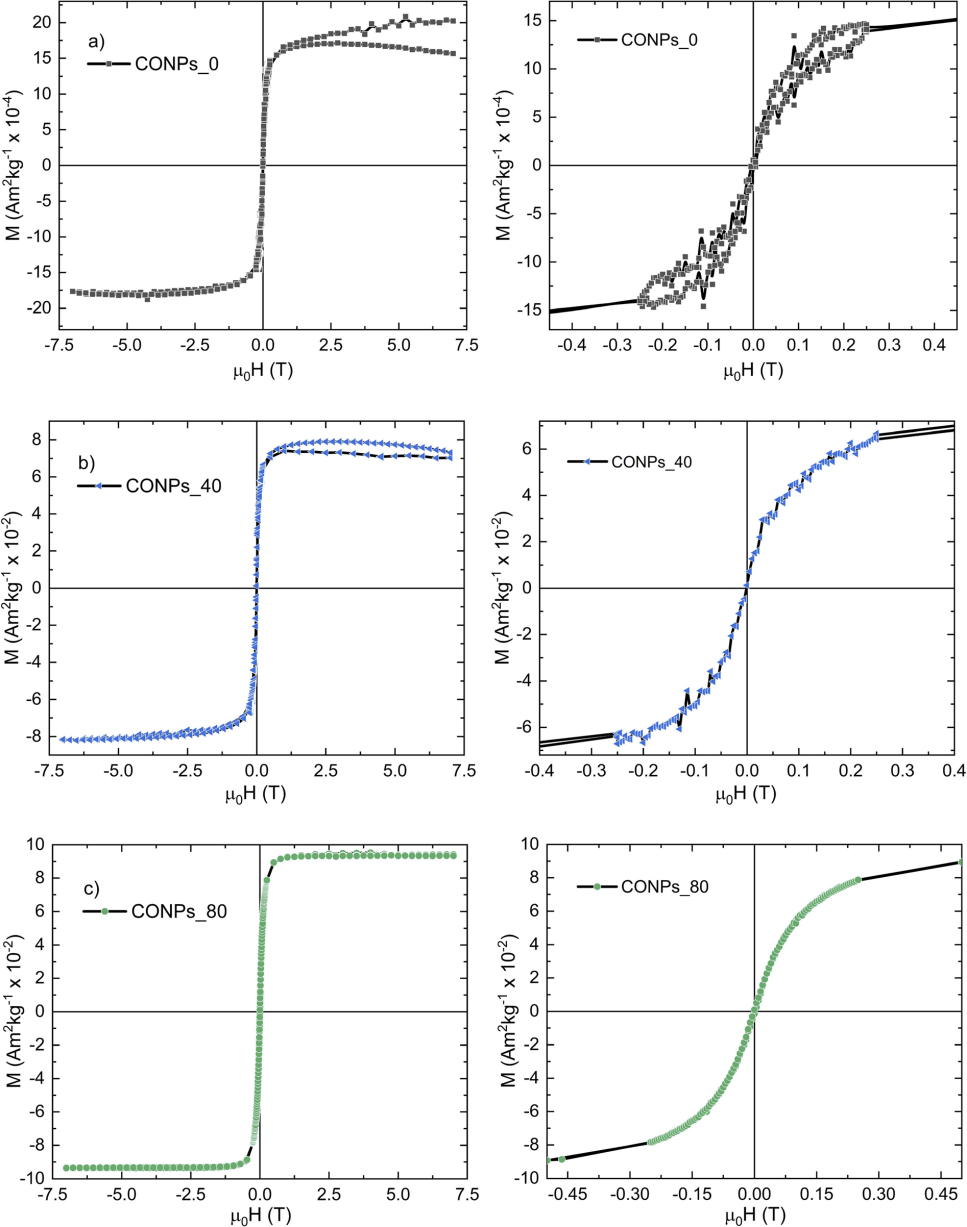
Magnetisation curves (M *vs. µ*_0_*H*) at 300 K for (a) CONPs_0, (b) CONPs_40, and (c) CONPs_80, measured using a SQUID magnetometer over ±7 T. The magnified panels on the right show the low-field region, highlighting the essentially superparamagnetic behaviour and the increase of saturation magnetisation with irradiation dose.

The CONPs_0 in Fig. 9 and CONPs_10 in S9 reveal the minor splitting of the upper branches with a higher saturation magnetisation (MS) in the second cycle. This may be explained by slow relaxation or field-induced conditioning effects of CeO_2_ NPs in a high magnetic field, given that the magnetic signal of the sample is close to the detection threshold of the measurement setup.

The magnetic hysteresis data for CONPs_10 sample are not included in Fig. 9 because incomplete drying by lyophilization led to residual solvent content, which affected the measured magnetisation. The observed magnetisation was lower than that of the CONPs_0 sample and, therefore, cannot be considered representative of the effects induced by irradiation. For completeness, these data in Fig. S9 will be provided in the SI in the revised manuscript.

The *M* − *µ*_0_*H* magnetization curves for CONPs_40 and CONPS_80 samples are shown in Fig. S8. After subtraction of their dia- and paramagnetic contributions, these curves show hysteresis loops with negligible coercivity and remanence typical for superparamagnetic NPs. Previous studies have shown that *M*_S_ values for CeO_2_ NPs vary widely depending on synthesis conditions and morphology.^14,79–83^ For instance, Uttara *et al.*^79^ reported a higher *M*_S_ of ≈0.006 A m^2^ kg^−1^ for 5.3 nm CeO_2_ nanocubes. In this context, the sample CONPs_0 with the size 2 ± 0.6 nm prepared by the thermal decomposition method exhibits a comparable *M*_S_ ≈ 0.002 A m^2^ kg^−1^. After irradiation, *M*_S_ increases to ≈0.08 and ≈0.09 A m^2^ kg^−1^ for CONPs_40 (3 ± 0.9 nm) and CONPs_80 (3 ± 0.9 nm), respectively, which places these values among the higher range reported for nanoceria of larger size.^14,79–84^ Once a sufficient irradiation time is reached, the defect-related magnetisation becomes strongly enhanced after irradiation and remains similarly high for the two highest-exposure samples that yielded reliable magnetic data. Wide-scan XPS spectra of the samples (Fig. S2 in the SI) show only Ce, O, and C (with minor Si contribution). Accordingly, the observed increase in *M*_S_ for CONPs_40 and CONPs_80 is consistent with an increased population of vacancy-related Ce^3+^ centres generated by irradiation and responsible for DIM in CeO_2_ nanocrystals. However, owing to dose uncertainties of ±30%, the observed trend is only correlational with the nominal irradiation time. While the absolute Ce^3+^ fraction is not quantified, the enhanced *M*_S_ is broadly consistent with the redox trends supported by XPS/EELS. Notably, optical disorder metrics (EU) and the apparent optical gap partially relax from CONPs_40 to CONPs_80, and XPS suggests a more Ce^4+^ -rich outermost surface at the highest dose. However, the similarly high *M*_S_ for CONPs_80 indicates that a substantial population of localised 4f^1^ electrons associated with Ce^3+^ centres persists in the nanoparticle interior and continues to dominate the room-temperature magnetic response.

## 4. Conclusion

We have established a modified thermal decomposition protocol in which reduced-pressure degassing is essential for reproducibly preparing ultrasmall CeO_2_ nanocrystals in the 2–3 nm range. These nanocrystals then served as a robust colloidal platform for post-synthetic treatment with a 16.5 MeV microtron electron beam. Although direct dosimetry was not available and the absorbed doses should therefore be regarded as nominal estimates, the irradiation time series clearly demonstrates that high-energy electron exposure can modify the defect landscape of ultrasmall nanoceria without destroying their crystalline fluorite-dominant cores.

Across the irradiation series, XPS and TEM indicate irradiation-induced changes in the Ce^3+^/Ce^4+^ ratio and oxygen-vacancy-related states, while HR-TEM confirms that the NPs remain crystalline even after the longest exposure. Surface-sensitive readouts show a non-monotonic response: the intermediate irradiation condition gives the strongest defect-related optical signatures, including the narrowest apparent optical gap and the highest Urbach energy, whereas the highest irradiation condition shows partial recovery of the optical response together with signs of near-surface reorganisation. A modest increase in the mean core diameter and broadening of the size distribution may contribute to the optical evolution, but these structural changes alone do not explain the observed trend as convincingly as the evolution of defect-related surface disorder.

In contrast to the partially recovering optical response, the room-temperature saturation magnetisation remains enhanced after irradiation. This suggests that defect centres generated by the electron beam are not restricted to the surface, but remain active within the NP volume even when the near-surface region undergoes partial reorganisation. Taken together, these results identify microtron electron irradiation as an effective post-synthetic route to defect-engineered ultrasmall nanoceria, capable of tuning surface-sensitive optical behaviour and volume-integrated magnetic response while preserving the crystalline nanocrystal framework.

## Author contributions

Z. Šiška: investigation, methodology, writing – original draft preparation, review and editing; T. Sojková: investigation, data curation, formal analysis; M. Sojka: conceptualisation, formal analysis, editing; P. Roupcová: investigation, data analysis, validation; M. Mihálik: magnetic measurements and analysis; K. Bukvišová: experimental support, sample preparation; D. Krishnan: experimental support; L. Šimoníková: experimental support; R. Gröger: investigation, data analysis, editing; N. Pizúrová: conceptualisation, project administration, funding acquisition, supervision, writing, review and editing.

## Conflicts of interest

There are no conflicts to declare.

## Supplementary Material

NA-008-D6NA00191B-s001

## Data Availability

All datasets that support the findings of this article are openly available at DOI: https://doi.org/10.5281/zenodo.18876429. Supplementary information (SI): characterisation data for CONPs_0, CONPs_10, CONPs_40, and CONPs_80, including DLS size distributions presented by number and volume together with TEM size statistics, wide-scan and valence-band XPS spectra with binding energies of the Ce 3d and O 1s spectra, UV-vis absorption spectra with corresponding Tauc plot and Urbach energy analyses, raw magnetic hysteresis loops measured at room temperature (300 K) for irradiated CONPs_40 and CONPs_80 samples, and magnetisation curves (M *vs. µ*_0_*H*) measured at 300 K for CONPs_10, including a magnified view of the low-field region. See DOI: https://doi.org/10.1039/d6na00191b.
