# Supplementary material for: Microtron electron beam enables post-synthetic defect engineering in ultrasmall ceria nanocrystals
Source: Nanoscale Adv. 2026 Jun 2;8(13):3666–79. doi: 10.1039/d6na00191b (PMC13267189; doi:10.1039/d6na00191b)
Supplement: NA-008-D6NA00191B-s001 [file NA-008-D6NA00191B-s001.pdf]

Supplementary Information (SI) for Nanoscale Advances.
This journal is © The Royal Society of Chemistry 2026

Supplementary information

Microtron electron beam enables post-synthetic defect engineering in ultrasmall ceria nanocrystals

Zuzana Šiška,[ab] Tereza Sojková,*[a] Martin Sojka,[a] Pavla Roupcová,[ab] Marián Mihálik,[c] Kristýna Bukvišová,[bd] Dileep Krishnan,[e] Lucie Šimoníková,[f] Roman Gröger,[a] Naděžda Pizúrová*[a]

[a] Institute of Physics of Materials and CEITEC IPM, Czech Academy of Sciences, Žižkova 513/22, 616 00 Brno, Czech Republic.

[b] Central European Institute of Technology - CEITEC, Brno University of Technology, Purkyňova 656/123, 612 00 Brno, Czech Republic

[c] Institute of Experimental Physics, Slovak Academy of Sciences, Watsonova 47, Košice 040 01, Slovakia

[d] Thermo Fisher Scientific Brno, Vlastimila Pecha 1282/12, 627 00 Brno, Czech Republic

[e] Thermo Fisher Scientific, Achtseweg Noord 5, 5651 GG Eindhoven, The Netherlands

[f] Masaryk university, Department of Chemistry, Brno, Kotlářská 267/2, 611 37, Czech Republic

*Corresponding Authors: Tereza Sojková (sojkova@ipm.cz)

S3.4 Nanoparticle size analysis by TEM and DLS

DLS analysis in Figure S1 reveals a gradual increase in the hydrodynamic diameter (D_H) of NPs with increasing irradiation dose. Both intensity-based and volume-based size distributions showed a clear shift toward larger particle sizes as the irradiation time increased from 0 to 80 minutes. CONPs_0 immediately after synthesis exhibits a narrow size distribution depicted in Table S1, indicating a well-dispersed nanoparticle population. After low-dose irradiation (CONPs_10), only a slight increase in D_H was observed, suggesting minimal structural or colloidal changes. However, more pronounced changes were observed for CONPs_40 and CONPs_80, with D_H values increasing significantly. This trend suggests particle growth or aggregation induced by irradiation. Notably, the particle distribution by intensity consistently showed slightly higher D_H values compared to distribution by number and volume (Table S1), which is expected due to the increased light scattering contribution from larger particles. This phenomenon has been widely reported in DLS analysis, where the intensity of scattered light increases with the sixth power of particle diameter, thereby amplifying the signal from even a small number of large particles or aggregates[1].

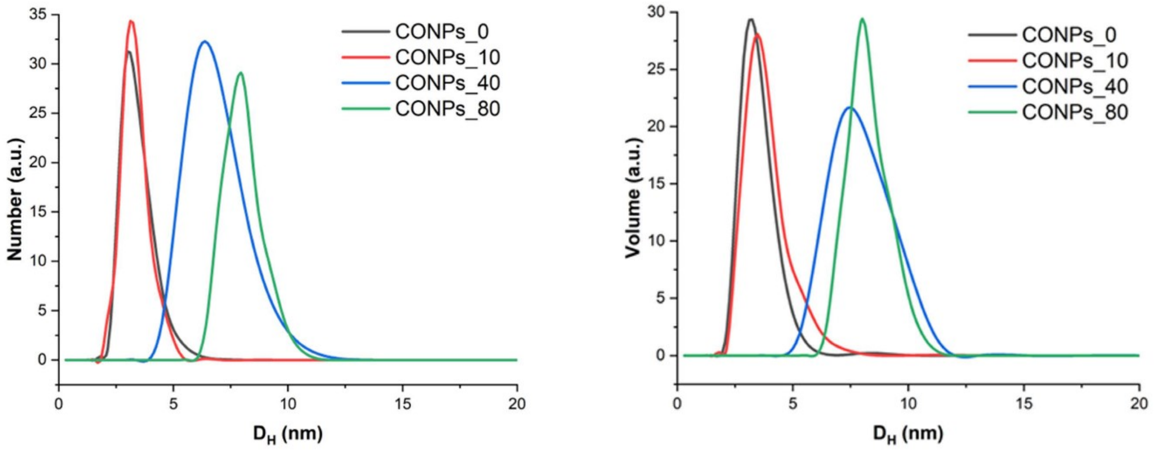

Figure S1. Histograms of hydrodynamic sizes (D_H) represented by number and volume for the sample CONPs_0 and irradiated samples CONPs_10/40/80.

Table S1. Summary of particle sizes from TEM, hydrodynamic sizes determined by DLS, number, volume for samples CONPs_0 – CONPs_80.

Sample	TEM size (nm)	Size by Intensity (nm)	Size by Volume (nm)	Size by Number (nm)
**CONPs_0**	2.1 ± 0.6	4.2 ± 1	3.5 ± 1	3 ± 0.5
**CONPs_10**	2.1 ± 0.6	5 ± 2	3.8 ± 1	3.4 ± 1
**CONPs_40**	2.8 ± 0.9	7.9 ± 4	8 ± 4	6.7 ± 5
**CONPs_80**	2.6 ± 0.9	10 ± 2	10 ± 2	8 ± 2

S3.5 XPS analysis of oxidation state and defects

In the following tables, we list the binding energy values of Ce 3d, O 1s. Table S2 shows the Ce 3d core-level spectra of CONPs_0/10/40/80. A characteristic set of multiplet-split doublets arises from the spin–orbit splitting of the 3d orbital into $3d_{5/2}$ (denoted as v) and $3d_{3/2}$ (denoted as u) components located at the corresponding energies. The energy is associated with the final state of Ce^{3+} ions ($4f^1$ configuration), with v^0/u^0 corresponding to the main peaks and v'/u' to their respective satellite structures. Peaks corresponding to Ce^{3+} (u', u^0, v', v^0) are listed. The characteristic peaks of the Ce^{4+} (u''', u'', u^0, v''', v'' and v^0) oxidation state ($4f^0$ configuration) include the binding energies due to final-state hybridisation effects between the O 2p and Ce 4f orbitals that was analysed in the CONPs_80 sample only. The binding energy of Ce^{4+} is u''' (917.22 eV), v''' (898.2 eV), u'' (907.6 eV), v'' (888.6 eV), u^0 (900.5 eV), and v^0 (883.7 eV).[2]

Table S2. Ce 3d binding energies for Ce^{3+} in the sample CONPs_0 (immediately after synthesis) and irradiated samples CONPs_10, CONPs_40 and CONPs_80.

**Sample**	Binding Energy (eV)			
	**Ce^{3+}**			
	u'	u^0	v'	v^0
**CONPs_0**	904.52	900.46	886.05	882.25
**CONPs_10**	904.37	900.28	885.71	880.07
**CONPs_40**	904.35	900.34	885.94	880.30
**CONPs_80**	904.31	900.28	885.86	882.14

In Table S3, the O 1s signals, deconvoluted into binding energies of adsorbate species of OA or defects (O-H), surface oxygen O-C bound to C-O of OA and Ce^{3+}-O, lattice oxygen bound to Ce^{4+} and Si^{4+} (O_L).

Table S3. O 1s binding energies showing contributions corresponding to lattice oxygen and surface species related to C-O, Ce^{3+}-O and Ce^{4+} oxidation states, consistent with the Ce 3d spectral features.

Sample	Binding energy (eV)		
	O-H	O-C (C-O/Ce^{3+}-O)	O_L (Ce^{4+}/Si^{4+})
**CONPs_0**	533.4	531.4	528.8
**CONPs_10**	533.7	532	529.8
**CONPs_40**	533.3	531.2	528.9
**CONPs_80**	533.7	531.9	529.2

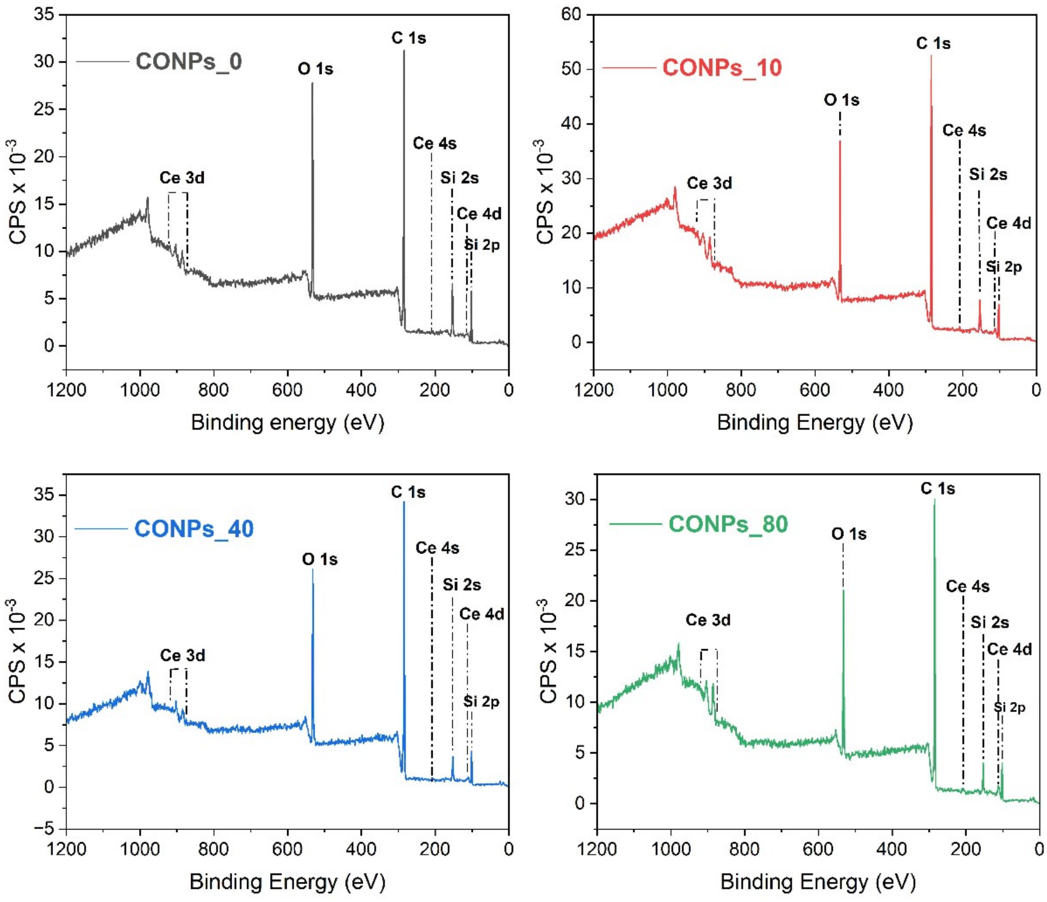

Figure S2. Wide-scan XPS spectra of sample CONPs_0 immediately after synthesis and irradiated samples CONPs_10, CONPs_40 and CONPs_80.

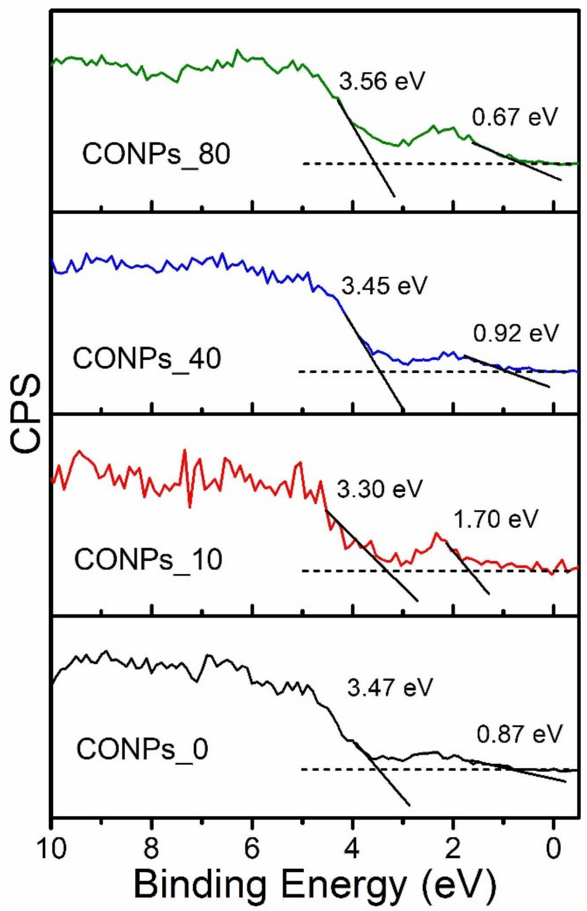

Figure S3. Valence band XPS (Al Kα) of CONPs_0, CONPs_10, CONPs_40 and CONPs_80. Electron irradiation redistributes intensity near the valence band edge and gives rise to additional low binding energy states, indicative of defect-related states within the band gap.

S3.6 Optical properties

The absorption coefficient (α) was used to determine the bandgap (E_g). It is derived using the Beer-Lambert law $\alpha = 2.303A/l$, which uses measured absorbance (A) and the optical length (l) of the absorbing medium. In the measurements, incident photons pass through a square cuvette with edge $l = 1\ cm$. To quantify irradiation-induced changes, the apparent optical band gap E_g was estimated from Tauc-type fits of the UV–Vis spectra (Fig. S4) by extrapolating the selected linear region to the energy axis.[3] Here, E_g is used as a phenomenological descriptor for comparing samples because the spectra exhibit a pronounced sub-gap tail, and the extracted intercept can depend on the assumed transition model.

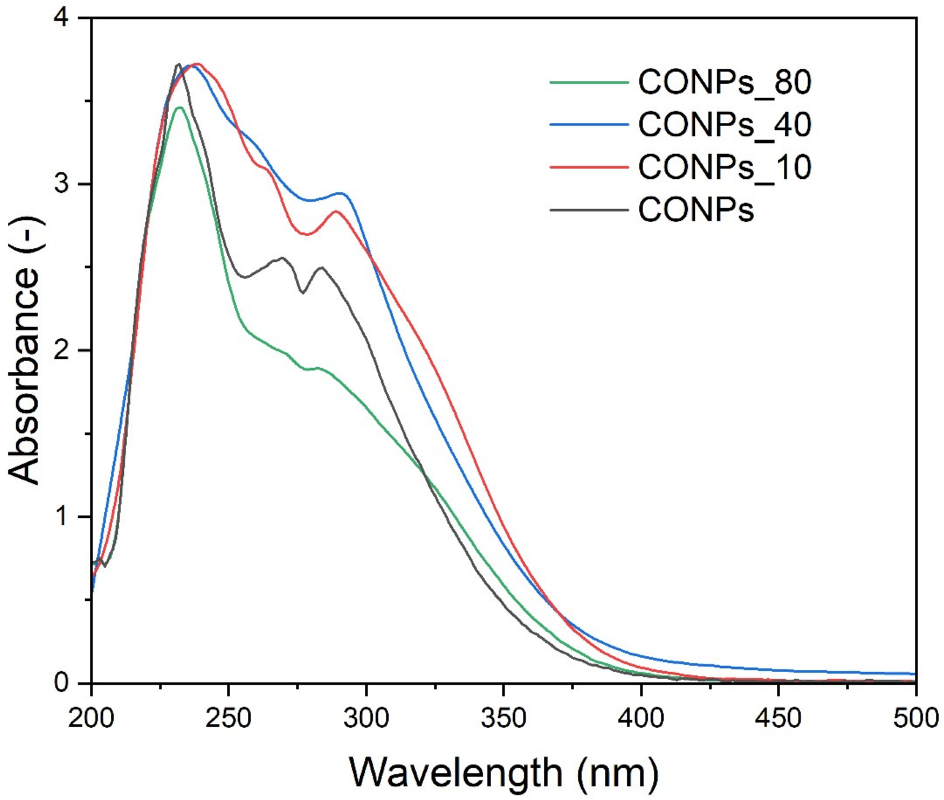

Figure S4. UV–Vis absorbance spectra of irradiated samples (CONPs_10, CONPs_40, and CONPs_80) and their comparison with the non-irradiated sample CONPs_0 after synthesis showing changes in the absorption edge caused by irradiation.

Tauc plot (amorphous or disordered materials):

The left panels in Figures S5 and S6 show fits of the absorption coefficient using different models. Only the red datapoints are considered for the linear regression. The right panels are numerical derivatives of experimental data, used to demonstrate the flatness of the data from which the fit is made.

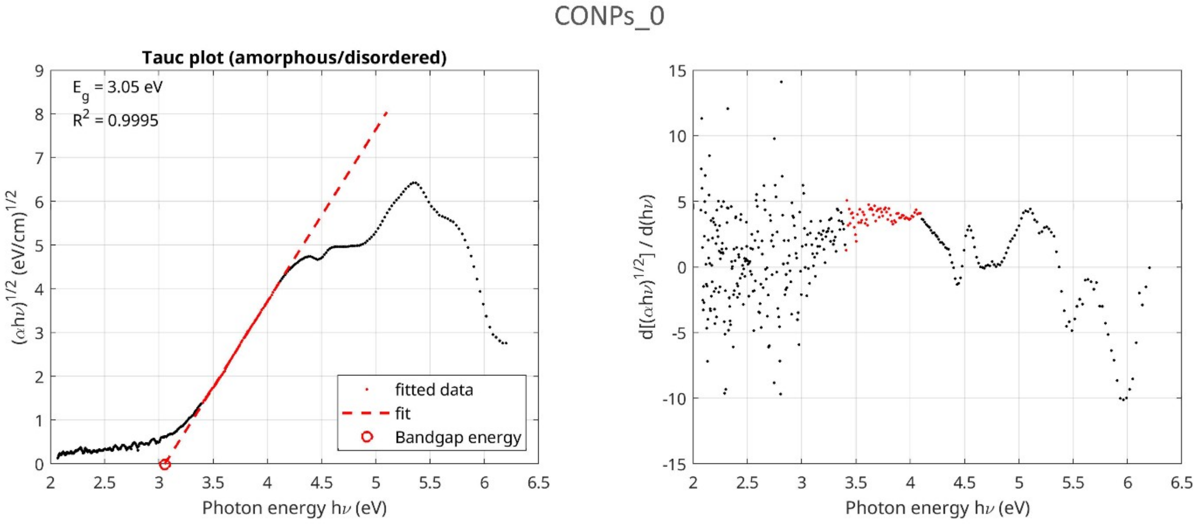

Figure S5. Left: Linear regression of the absorption coefficient for the as-synthesised CONPs_0 sample based on red data points. Right: Numerical derivatives illustrating the flatness of the fitted region.

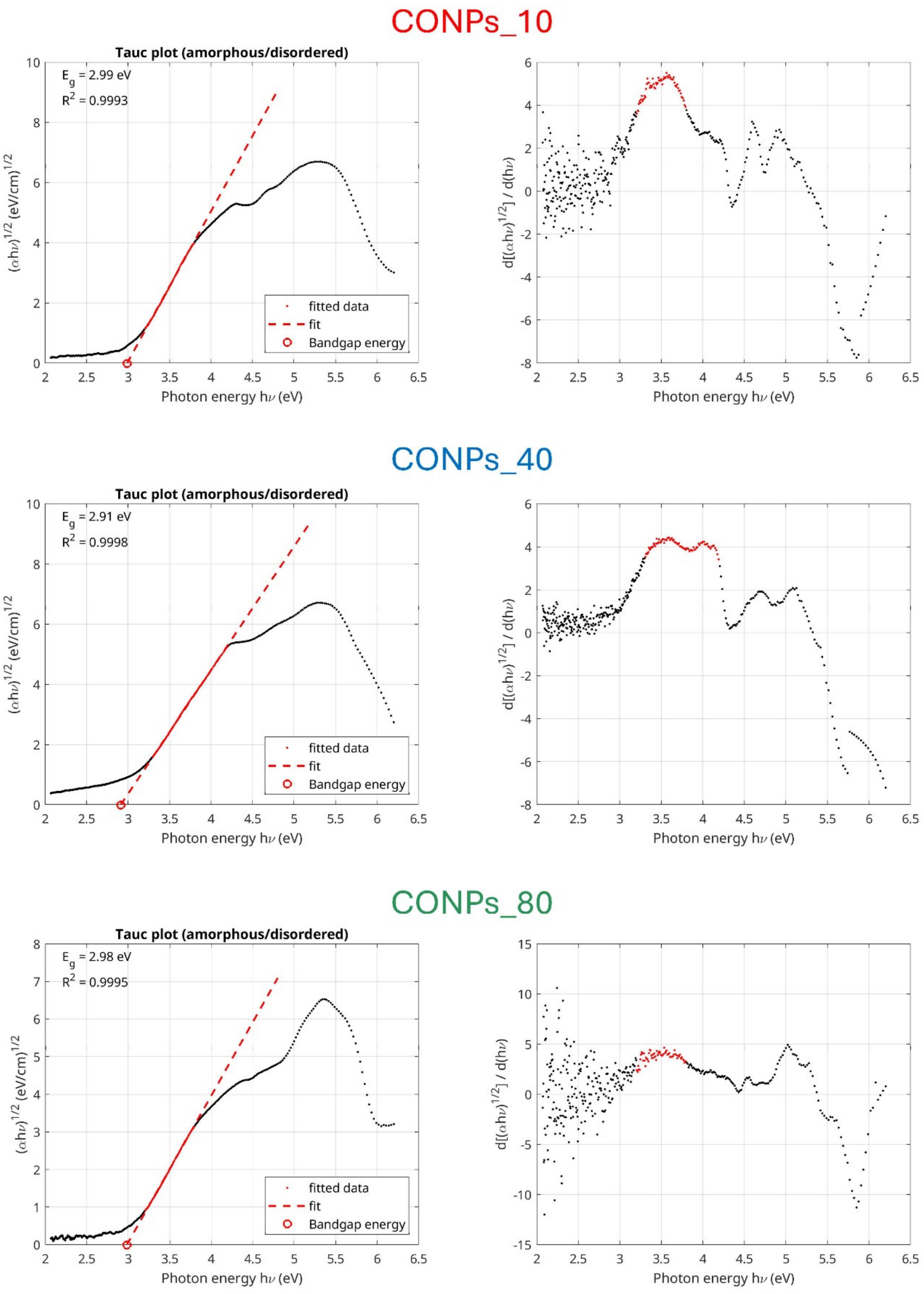

Figure S6. Left: Linear regression of the absorption coefficient for irradiated samples CONPs_10, CONPs_40, and CONPs_80 at different exposure times based on red data points. Right: Numerical derivatives illustrating the flatness of the fitted regions.

Urbach energy:

Below the bandgap, the absorption coefficient of direct-gap semiconductors exhibits an exponential (Urbach) tail in Figure S7. In this region, the absorption coefficient can be approximated as

$$\alpha = \alpha_0 \exp\left(\sigma \frac{E - E_g}{kT}\right)$$

where α_0 and σ are fitting coefficients. The Urbach energy, $E_U = kT/\sigma$, depends on temperature which is 300 K in our measurements. The Urbach energies for four samples were determined from the sub-bandgap region using the optical band gaps estimated from the corresponding Tauc plots

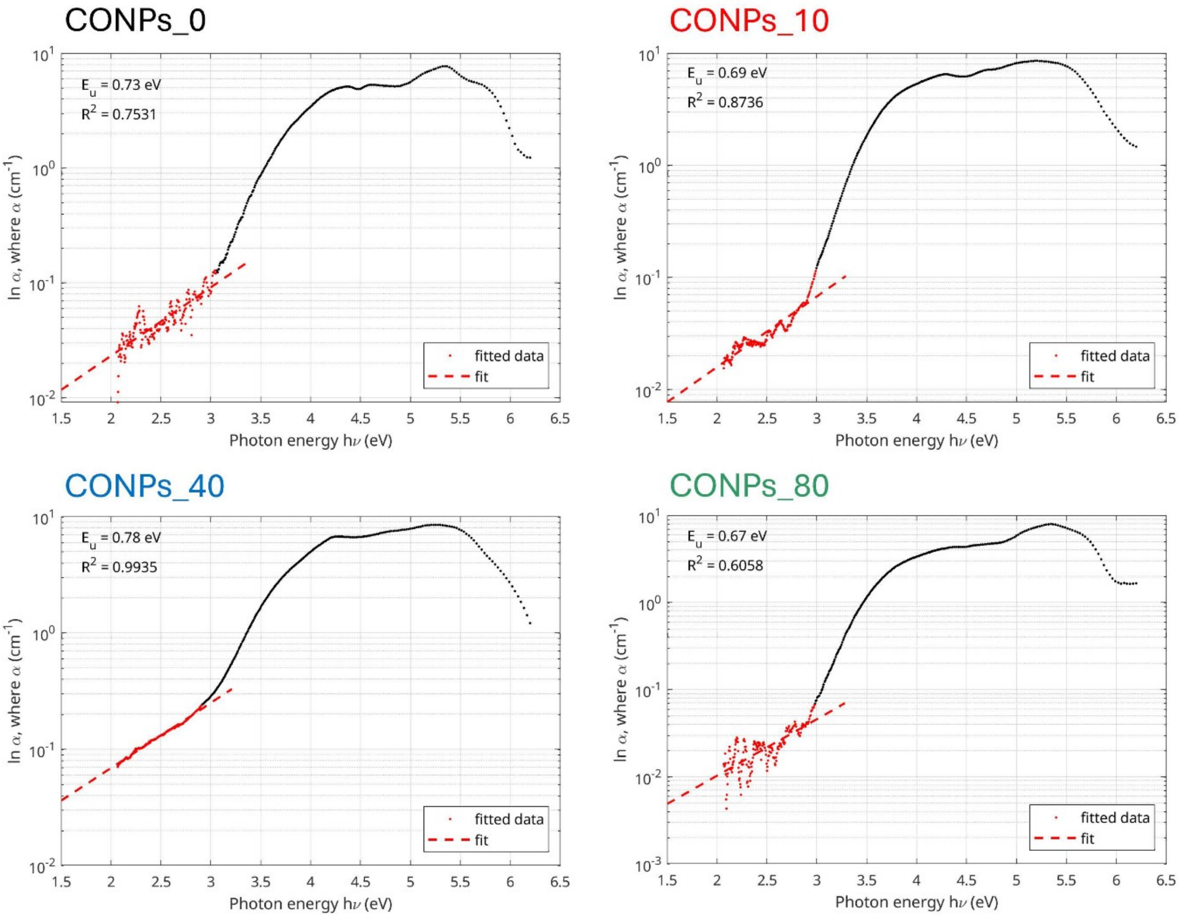

Figure S7. Determination of the Urbach energies for as-synthesised and irradiated samples CONPs_0, CONPs_10, CONPs_40, and CONPs_80. The plots show the linear regression (dashed

red lines) applied to the exponential absorption tails (red data points). The Urbach energy for each sample is derived from the slope of the fit in the region below the bandgap. The numerical values of E_U and the corresponding coefficients of determination (R^2) are indicated in each panel.

S3.7 Magnetic properties

The $M - \mu_0 H$ loops in Figure S8 were recalculated in SI units as a dependence of magnetisation (Am^2/kg) on the applied magnetic field (T) where the magnetisation values were normalised to the mass of the samples. Measurements were performed at room temperature in the applied magnetic field up to ±±7 T. The measured signal included diamagnetic and paramagnetic contributions, which were subtracted prior to analysis of the magnetic response associated with Ce^{3+} centres.

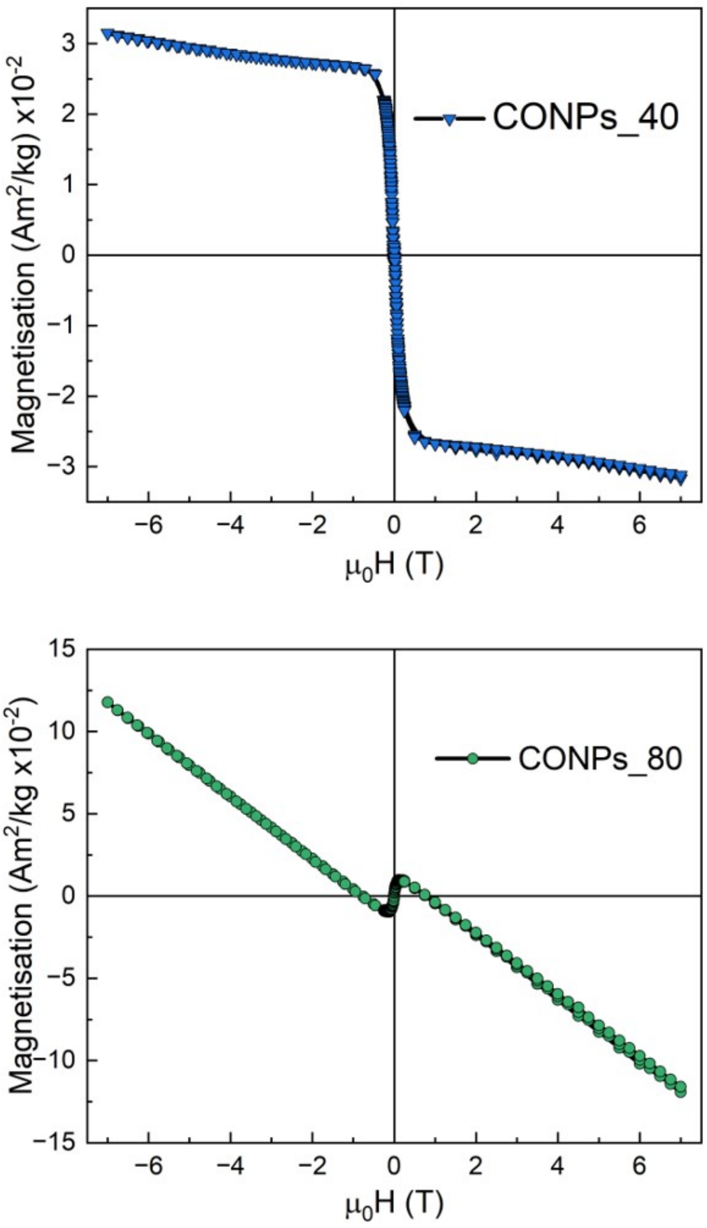

Figure S8: Raw data of hysteresis loops at room temperature (300K) after irradiation dose for samples CONPs_40 and CONPs_80.

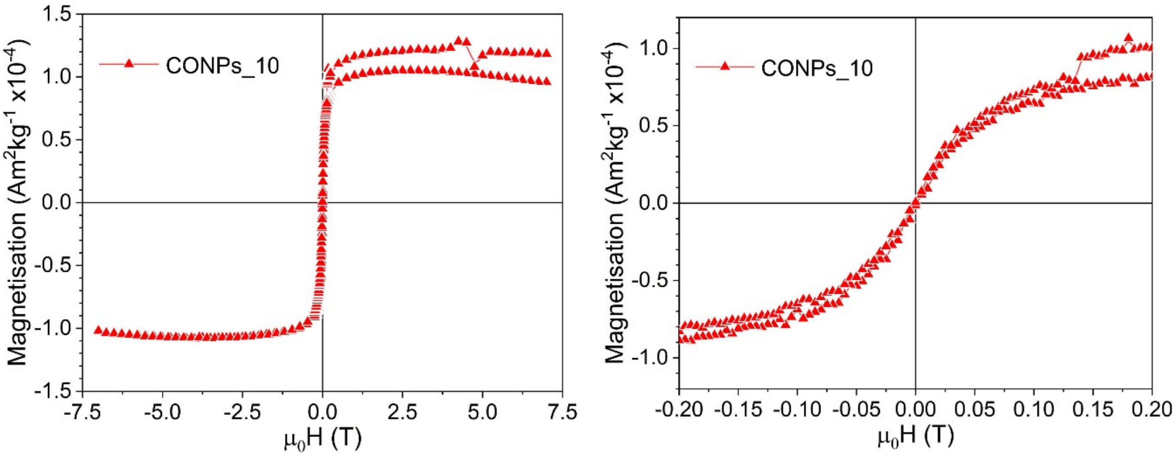

Figure S9: Magnetisation curves (M vs μ_0H) at 300K for CONPs_10, measured using a SQUID magnetometer over ±7 T. The magnified panel on the right show the low-field region, highlighting the essentially superparamagnetic behaviour.

References

1 J. Stetefeld, S. A. McKenna and T. R. Patel, *Biophys Rev*, 2016, **8**, 409–427.

2 R. Eloirdi, P. Cakir, F. Huber, A. Seibert, R. Konings and T. Gouder, *Applied Surface Science*, 2018, **457**, 566–571.

3 M. Fox, *Optical properties of solids*, Oxford University Press, Oxford ; New York, 2nd ed., 2010.
